# Supplementary material for: Brain-Derived Neurotrophic Factor (BDNF) as a Marker of Physical Exercise or Activity Effectiveness in Fatigue, Pain, Depression, and Sleep Disturbances: A Scoping Review
Source: Biomedicines. 2025 Jan 31;13(2):332. doi: 10.3390/biomedicines13020332 (PMC11853410; doi:10.3390/biomedicines13020332)
Supplement: Supplementary file 1 [file biomedicines-13-00332-s001.zip › Table S1 - Search String Table.pdf]

Key Words

Database: PubMed – MEDLINE and CINAHL Plus Full Text

Limit to: Published in English

Search date:

Population: Adults 18 years and older

Intervention: Exercise or Physical activity

Comparison: No comparison

Outcomes: Brain-Derived Neurotrophic Factor OR BDNF, Sleep, Depression, Pain, Fatigue

Table S1. *Search String*

Database (Search time)	Search	Search String
**PubMed (847 reports)**	1	("exercise"[MeSH Terms] OR "exercise"[All Fields] OR "exercises"[All Fields] OR "exercise therapy"[MeSH Terms] OR ("exercise"[All Fields] AND "therapy"[All Fields]) OR "exercise therapy"[All Fields] OR "exercising"[All Fields] OR "exercise s"[All Fields] OR "exercised"[All Fields] OR "exerciser"[All Fields] OR "exercisers"[All Fields] OR ("exercise"[MeSH Terms] OR "exercise"[All Fields] OR ("physical"[All Fields] AND "activity"[All Fields]) OR "physical activity"[All Fields])) AND ("brain derived neurotrophic factor"[MeSH Terms] OR ("brain derived"[All Fields] AND "neurotrophic"[All Fields] AND "factor"[All Fields]) OR "brain derived neurotrophic factor"[All Fields] OR ("brain"[All Fields] AND "derived"[All Fields] AND "neurotrophic"[All Fields] AND "factor"[All Fields]) OR "brain derived neurotrophic factor"[All Fields] OR ("brain derived neurotrophic factor"[MeSH Terms] OR ("brain derived"[All Fields] AND "neurotrophic"[All Fields] AND "factor"[All Fields]) OR "brain derived neurotrophic factor"[All Fields] OR "bdnf"[All Fields])) AND ("sleep"[All Fields]

		OR "pain"[All Fields] OR "depression"[All Fields] OR "fatigue"[All Fields])
# 1 with filters<br>**(287 reports)**	2	Limit search to English and Human studies
**CINAHL plus**<br>**(110 reports)**	1	(Exercise or Physical activity) AND (brain-derived neurotrophic factor or bdnf) AND (fatigue OR pain OR depression OR sleep) **Expanders -** Apply related words; Apply equivalent subjects<br>**Search modes -** Find all my search term
#1 with filters<br>**(55 reports)**	2	Limit English, limit human
