# Supplementary material for: Brain-Derived Neurotrophic Factor (BDNF) as a Marker of Physical Exercise or Activity Effectiveness in Fatigue, Pain, Depression, and Sleep Disturbances: A Scoping Review
Source: Biomedicines. 2025 Jan 31;13(2):332. doi: 10.3390/biomedicines13020332 (PMC11853410; doi:10.3390/biomedicines13020332)
Supplement: Supplementary file 1 [file biomedicines-13-00332-s001.zip › Table S2- Detail intervention, measures, key findings.pdf]

Supplementary Table S2. Interventions, Measures, and Key Findings

Author, Year of publication	Exercise or Physical Activity		Comparison	BDNF Sample/ Detection method	Symptoms-Measures	Result	
	Frequency; duration	Intervention				Change in BDNF	Symptoms vs BDNF
			**Adults with Neurological Disorders**				
1. Amato et al., 2021 [52]	12 weeks (2 x/week); 90 mins/session	Stretching exercise: lactate threshold training (combined balance and stretching exercise to produce a modest amount of lactate)	Pre vs. Post vs. 9 months follow up	Plasma/ ELISA	Fatigue-VAFS	The BDNF level increased significantly immediately after the intervention program but not at 9-month post intervention follow up	Immediately after the intervention, fatigue decreased and BDNF level increase significantly
2. Azevedo et al., 2022 [53]	One session; 60 mins	Aerobic exercise- Walking on a treadmill with increasing speed during the first 15 min to reach 60-65% Max HR. Continue with the same intensity (15 min) and decrease speed for cool down (5 min)	Participants with vs. without depression and PD participants with vs. without fatigue	Serum/ ELISA	Depressive symptoms-BDI Fatigue- PFS	mBDNF level increased after the exercise regardless groups.	Symptoms (fatigue and depression) were used for grouping. Exercise increase mBDNF level regardless groups
3. Bansi et al, 2013 [54]	3-week (5x/week); 20 mins	Aerobic exercise: Cycling on an aquatic bike (Training intensity: 70% Heart Rate peak (60% VO_2 peak) (n=28)	Cycling on an ergometer (Training intensity: 70% Heart Rate peak (60% VO_2 peak) (n=24)	Serum/ Multiplex flow cytometry	Fatigue- The multidimensional FSMC	No significant differences of BDNF between the land vs water ergometer groups. In the water group, BDNF significantly increased after the intervention	The increasing of BDNF were observed, fatigue was not changed after the intervention.
4. Bartlett et al., 2020 [55]	9 months (2x/week); 60 mins	Multimodal exercise: Supervised aerobic, resistance and endurance training combined with other interventions in rehabilitation program. (n=18)	Usual Care Control (n=11)	Serum/ ELISA	Sleep-PSQI, sleep diary, ESS Anxiety, and depression-HADS	The BDNF level slightly increased from baseline in the intervention group.	Changes in BDNF level and symptoms after the intervention were not significant

5. Devasahayam et al., 2020 [56]	One session	Aerobic exercise: Maximal GXT on a TBRS- 80 SPM for 2 minutes, increasing the load by one level every 2 mins until exhaustion or level 10. (n=14)	Age/sex matched healthy control (n=7)	Serum/ ELISA	Fatigue-FSS HRQoL-SF-36)	Serum BDNF level increased after the intervention but not significant.	In MS group, higher elevation in BDNF was significantly correlated with walking speed and lower vitality (measured by SF-36)
6. Belchior et al., 2017 [57]	8-week (2x/week); 10-30 mins/session	Aerobic exercise: Walking on a treadmill- Start with 10 minutes of walking at baseline velocity on a flat treadmill. Every week, increase by 5 minutes until reaching 30 minutes. (n=10)	Usual Care Control (n=12)	Plasma/ NA	Depression-GDS HRQoL-SF-36 cognitive function-MMSE	No significant change in BDNF level post-intervention changes.	Association between BDNF and symptoms was not investigated
7. Harro et al., 2022 [58]	20-week (2-3x/week); 60-65 mins/session	Aerobic exercise: NW training—supervised NW exercise training (6 week) and independent NW (14 weeks)	before, post supervised phase and 3 month follow up	Serum/ ELISA	Fatigue-PFS	BDNF level increased significantly at 3 month follow up from baseline and post supervised phase	No significant change in fatigue. The association between BDNF level and fatigue was not studied
8. Landers et al., 2019 [59	8 weeks (3-4x/week); 90 mins/session	Multimodal exercise: High-intensity multimodal exercise boot camp (HIBC)- Multimodal exercise-moderate to high intensity aerobic, strength training, balance training, and active rest and stretching (n=13)	Low-intensity, Control group- 60 mins of aerobic, strength, balance, and stretching (n=11)	Serum/ ELISA DNA/ Genotyping	Fatigue-PFS Depression-BDI QoL-Parkinson's Disease Questionanaire-39	BDNF levels significantly increased post-intervention but decreased at the 6-month follow-up compared to baseline in both HIBC and control groups.	Fatigue decreased significantly post intervention only in the HIBC group. The association between BDNF level and symptoms was not investigated.
9. Ozkul et al., 2018 [60]	8-week; 105 min/session	Multimodal exercise: combined aerobic and Pilates exercise- a) aerobic training: walking on a treadmill to reach 60-80% of maximum Heart; b) Pilates training after 15 min rest. (n=18)	Usual care control (n=18) vs. Healthy volunteers (n=18)	Serum/ ELISA Serum	Fatigue-FSS	The BDNF level increase post intervention in both groups but was significant only in the exercise group.	Fatigue severity decreased significantly only in exercise group but increase in the control group. The association between BDNF and symptoms was not investigated.

10. Liu et al., 2020 [61]	4-week (5x/week)	Strength training: isotonic weight training machines: 2 sets of 12 reps of a weight 40-50% 1 Repetition Maximum (n=30)	Aerobic (stationary bicycle training): paddle rate at the intensity of 5-6 on a 10-scale of perceived exertion for 30 min (n=31)	Serum/ ELISA	Cognitive function- MMSE and MoCA Depression- GDS	The BDNF level increase in both groups but not significant	Cognitive function improved significantly for both exercise groups. Both depression and BDNF level were not significantly change for both groups
11. Zhang et al., 2023 [62]	12-week (3x/week); 60 min/session	Multimodal exercise: Traditional Chinese Exercise (TCE) + Rhythm Training Group (RTG)- joint stretching, aerobic exercise and acupoint massage (n=14)	Walking Group- Relaxation phase (10 min) and 3 km walk (WG) (n=14)<br><br>Education Control group (n=14)	Serum/ ELISA	Depression- HDS	BDNF level increased in both TCE+RTG and WG groups but significantly decreased in the control group	Participants in both exercise groups (TCE+RTG and WG groups) showed significant reduction of depression score and increasing BDNF level at 12 weeks
			**Adults with chronic conditions**				
12. De Araujo et al., 2019 [63]	8-week (3x/week); 90 min/session	Multimodal exercise: Treadmill endurance (30 min at 60% 6MWT, progress by dyspnea) and muscular training: 2 sets of 10-15 reps for lower limbs, 2 sets of 2 minutes for upper limbs.	Pre vs. Post 1st session vs. Pre 24th session	Plasma/ ELISA	Depression- BDI, HRQoL- mMRC Dyspnea- SGRQ	Plasma BDNF level significantly decreased immediately after the first exercise session.	Dyspnea decreased significantly. No significant change of the depression score. Change in plasma BDNF was not related to change in depression and dyspnea
13. Deus et al., 2021 [64]	6-month (3x/week); 60 min/session	Strength training: Resistance training (RT)immediately before each dialysis session (n=81)	Usual care control (CTL) (n=76)	Serum/ ELISA	Depression- BDI, HRQoL- SF-36	BDNF increased for RT group but decreased for CTL group.	Depression decreased, and vitality and emotional well-being improved in the exercise group. High BDNF was linked to lower depression and better well-being.
14. Gomes et al., 2014 [65]	12-week (3x/week)	Aerobic exercise: Acute (2 min at 1 mph, 18 min at 2 mph, 30 min rest) following by chronic: 40-65 min aerobic exercise (5 min warm-up/cool-down), 30 min treadmill at 70% HRmax, increasing to 80% HRmax by week 8 or 12.	Pre-post intervention	Plasma/ ELISA	Pain-VAS for pain	Acute exercise: BDNF increases immediately and lasts up to 30 min during recovery.<br><br>Chronic exercise: BDNF significantly increases after 12 weeks compared to baseline.	Pain decreased significantly while the BDNF level increased significantly after the 12 weeks exercise compared to baseline.

15. Jablochkova et al., 2019 [66]	15-week (2x/week); 60 mins/session	Strength training: Resistance training: 10 min warm-up and stretching. Progress from 40% to 80% of 1RM, with 15-20 reps to 5-8 reps, 1-2 sets, and 1 min rest. (n=41)	Relaxation therapy: 25 min guided relaxation (n=34)<br><br>Healthy Volunteers (n=25)	multiplex electrochemiluminescence assay panel Plasma	Pain- VAS<br>Anxiety and Depression-HADS<br>HRQoL-SF-36 Quality of Life,<br>Fibromyalgia-FIQ<br>Fatigue-MFI	FM participants had higher BDNF than controls.<br><br>BDNF increased post-intervention in both groups, but not significantly.	Pain and fatigue decreased significantly in the exercise groups.<br><br>No significant association between BDNF change and symptoms.
16. Lee et al., 2014 [67]	12-week	Aerobic exercise: 50 minutes of moderate aerobic exercise in weight reduction program	Pre-post intervention: Participants were grouped by BDNF change (minor vs. greater)	Serum/ ELISA	Depression-Zung Self-Rating Depression Scale	The BDNF level increased significantly after the intervention	Depression score decreased, but not significantly. Larger BDNF change linked to smaller depression decrease.<br>Significant reduction in depression was seen in the greater BDNF change group.
17. Ribeiro et al., 2021 [68]	6 weeks (3x/week)	Others: Whole-Body Vibration Training (WBVT)- 3-11 min of dynamic squatting on a synchronic vibrating platform (FitVibe®ExcelPro, GymnaUniphy, Belgium). (n=17)	Usual care Control (n=15)	Plasma/ ELISA	Fibromyalgia Symptoms-FIQ and VAS<br>Sleep-PSQI<br>Depression-BDI	BDNF level increase significantly in the intervention group but decrease in the control group	The intervention group showed significant reduction of pain, sleep problem, and depression and increasing BDNF level
18. Žlibinaitė et al., 2020 [69]	6 months (3x/week); 50 mins/session	Aerobic exercise: supervised aerobic exercise training on cycle ergometry to achieve 60-70% of maximum HR (n=13)	Usual Care Control (n=13)	Serum/ ELISA	Mood State-Brunel Mood Scale	BDNF level decreased after the program. The control group had a larger reduction of BDNF level.	No significant changes in exercise group. In control group, depression and confusion increased significantly and BDNF level decreased.

19. Maguire et al., 2023 [70]	One session; 80 mins	Aerobic exercise: Moderate intensity cycle-ergometer aerobic training (MI-ET) and upper limb robotic or sensor-based motor training (RT) (n=10)	Low-intensity gait and balance circuit training (LI-CT) (n=7)	Serum/ ELISA	Balance measured with the Mini BESTest Fatigue-FSS	Serum BDNF levels showed no significant change from pre to post-training in either group.	Change in BDNF levels was not related to changes in balance and fatigue
**Adults with Cancer**							
20. Cartmel et al., 2021 [48]	6-month	Aerobic exercise: Home based moderate-intensity aerobic exercise- Weekly phone consultation with certified cancer exercise trainer to increase the PA to 150 min/week. (n=74)	Attention control health education- Weekly telephone attention control health education. (n=70)	Serum/ ELISA	Depression- CES-D	Free-BDNF level decreased but the total BDNF level increased after the program compared to baseline.	Depression decreased in the exercise arm.<br><br>Greater decline of depression score associated with a greater increase in free-BDNF (weak association) but not the total BDNF
21. Hartman et al., 2019 [49]	12 weeks	Physical activity: Personalized physical activity- One in-person visit to set activity goals, two phone calls, and emails every 3 days with reminders and content. (n=43)	Wellness Contact Control Group- Health education through e-mails every 3 days (n=44)	Plasma/ High-sensitivity immunoassay	Anxiety, depression, fatigue, physical function- PROMIS Cognitive functions- SDT	No between-group differences in changes in BDNF.	The exercise group had greater reductions in anxiety. BDNF mediation on exercise effects on cognition was not studied due to the lack of significant effect on BDNF levels
22. Miklja et al., 2022 [71]		No intervention.	Low tolerance (Light activity daily) vs. High tolerance (Moderate to high activity for 10+ min/day.)	Plasma/ Circulating BDNF- ELISA DNA/ $BDNF^{val66met}$ polymorphism- PCR	Pain, sleep, fatigue, depression, and anxiety- PROMIS version 1.0	Glioma patients with high exercise tolerance had a lower mean plasma BDNF than low exercise tolerance participants.	High exercise tolerance reported higher quality of life. However, the amount of exercise and symptoms were not related to the BDNF polymorphism and circulating BDNF level

23. Zimmer et al., 2018 [50]	8-month	Multimodal exercise: Personalized exercise recommendation- 3-week rehabilitation to set activity goals (9-15 MET h/week), with adjustments at 1 week, 4, and 8 months. One phone follow-up after 1 month.	pre-post intervention	Serum/ ELISA	Fatigue- MFI-20 Cognitive function- EORTC QLQ-C30	BDNF level significantly increased after 8 months.	Fatigue decreased, self-perceived cognitive function increased and BDNF increased significantly after 8 months
**Older Adults**							
24. Gmiat et al., 2018 [72]	12-week (3x/week); 60 mins/session	Aerobic exercise: Beginners (first time) NW (n=11)	Advanced group (regular NW for more than 4 years) (n=24)	Serum/ ELISA	Depression- BDI Cognition- D2 test of attention, Trial Making Test A&B<br><br>HRQoL-SF-36	The BDNF level increase post 12-week NW among participants in advanced group but decrease among participants in beginners' group	The advanced group showed improved depression, quality of life, and cognition, with no link between symptoms and BDNF change
25. Pereira et al, 2013 [73]	10-week (3x/week); 60 mins/session	Strength training: Muscle strength training at 50% of 1RM, then adjusted after 2 weeks to 75% of 1RM.<br>(n=229)	Aerobic exercise training-Walking and free exercise to maintained at 65-80% of the age maximum HR (n=222)	Plasma/ ELISA	Depression- GDS	The BDNF level increase significantly post intervention only in the strengthening group.	Depression decreased significantly post intervention for both groups. The effect of the exercise was not mediated by the BDNF level.
26. Ruiz et al., 2015 [74]	8-week (3x/week); 40-45 min/session	Multimodal exercise: Structured, supervised light to moderate intensity exercise that consisted of Aerobic on cycle ergometer, strength resistance and stretching exercise training. (n=20)	Standard Care (n=20)	Serum/ Quantitative Immunoassays	Depression- GDS Cognition- MMSE	The BDNF increase after the program in the exercise group but not significant.	No significant relationships among change in BDNF, depression and cognitive function.
27. Yeh et al., 2015 [75]	3 months (3x/week); 50 min/session	Aerobic exercise: Aerobic exercise with music for 64% maximal HR (n=41)	Usual Care (n=26)	Serum/ Multiplex	Depression- BDI	The BDNF level increase significantly after the program in the intervention group	The intervention group showed reduced depression scores and increased BDNF levels, but the BDNF-depression link was not examined.

28. Takahashi et al., 2019 [51]	8 weeks	Physical activity: Increasing PA above usual lifestyle under free-living condition (n=19)	Usual care control (n=19)	Serum/ ELISA	Depression-GDS	The serum BDNF increased significantly in the active group.	Depression score decreased for both groups but not significant. The association between BDNF and depression was not studied.
29. Vedovelli et al., 2017 [76]	3-month (3x/week); 60 min/session	Multimodal exercise: Combination of aerobic exercises (e.g., walking, cycling) and muscle-strengthening activities (e.g., resistance training using body weight or weights). (n=20)	Usual care Control (n=9)	Serum/ ELISA	Depression-BDI<br><br>Anxiety-BAI	BDNF level increased significantly at 1 month and 3-month post intervention	Depression and anxiety significantly reduced and BDNF level increased at 1-month and 3-month post intervention in the exercise group
			**Healthy young adults**				
30. Cahn et al., 2017 [77]	3-month	Stretching training: Yoga and meditation retreat with daily mindfulness, yoga, breath-focused and non-doing meditation, and a vegetarian diet.	Pre vs. post intervention	Plasma/ ELISA	Depression, Anxiety and Somatic symptoms-Brief Symptom Inventory-18	BDNF level increased after a 3-month intervention.	Self-reported anxiety and depression decreased, while BDNF increased after the 3-month program, with higher BDNF levels linked to reduced anxiety.
31. Cullen et al., 2020 [78]	One session; 2 hours	Aerobic exercise: Submaximal cycling (45 min) to reach 60% VO2 max follow by self-paced maximal effort (15 min) and recovery (30 min)	Three sleep conditions: control (sleep 7–9 hours), partial deprivation (sleep 4 hours), and full deprivation (24 hours no sleep).	Plasma/ ELISA	Fatigue, Mood State - Modified Profile of Mood States questionnaire	BDNF level increased from baseline during and after exercise	Exercise during different types of sleep deprivation did not impact BDNF levels. BDNF was associated with liveliness but was not influenced by the type of sleep deprivation.
32. Piacentini et al., 2016 [79]	12 weeks (3 times/week); start at 20 mins and increased by 5 mins	Aerobic exercise: Intensified training- increase time and intensity cycle training by 70% from the normal training	Normal training-normal volume and intensity of cycle training. Recovery training-low intensity.	Plasma/ ELISA	Mood State POMS-24	BDNF level increased post intervention but no significant different among the 3 training conditions	Exercise induced mood disturbance was not associated with a change in plasma BDNF concentrations

33. Suzuki et al., 2014 [80]	9 weeks	Multimodal exercise: Military training- military-style physical exercises, including endurance running, strength training, obstacle courses, combat drills, and fatigue-inducing activities, with extended periods of sleep deprivation.	Before, during and after training	Plasma/ ELISA	Fatigue- VAS Sleep-Wrist motion and pulse wave interval	Plasma BDNF concentration significantly decreased.	Fatigue increased during training but decreased afterward. No significant link was found between BDNF changes and symptoms (fatigue, sleep, stress).
34. Verbickas, et al., 2017 [81]	One session	Aerobic exercise: Sprint Interval exercise (SIE): 12 bouts of 5-second all-out cycling sprints with 3 minutes rest between each. The resistance set at 7.5% of body weight. (n=10)	Stretch-shortening exercise (SSE): 200 drop jumps from height of 0.5 m and immediate maximum rebound with a 30-second interval between each jump. (n=10)	Serum/ ELISA	Fatigue-isometric torque and central activation ratio	BDNF increased at 2 min after SIE and decreased at 24 hours after SIE and SSE.	Associations were found on high baseline BDNF level with smaller change of central fatigue.<br><br>Larger change in BDNF 24 hours associated with a lower central fatigue
35. Verbickas, et al., 2018 [82]	One session	Others: High-volume drops jump exercise- 200 intermittent drop jumps	Baseline, immediately after 100 and 200 drop jumps, and then at 1 h and 24 h post-exercise	Serum/ ELISA Serum	Neuromuscular fatigue-isometric torque and central activation ratio Pain-VAS	Serum BDNF was decreased by 1 hour and 24 hours after exercise.	Change in BDNF levels was not associated with changes in peripheral fatigue or central fatigue

Note: BDNF, Brain-Derived Neurotrophic Factors; ELISA, Enzyme-Linked Immunosorbent Assay; VAFS, Visual Analogue for fatigue scale; HR, Heart Rate; PD, Parkinson's Disease; BDI, Beck Depression Inventory; PFS-Parkinson Fatigue Scale-16; mBDNF, Mature isoform BDNF; FSMC, Fatigue Scale for Motor and Cognitive Function; PSQI, Pittsburgh Sleep Quality Index; ESS, Epworth Sleepiness Scale; GXT, graded exercise test; TBRS, total body recumbent stepper; SPM, steps per minute; FSS, Fatigue Severity Scale; HRQoL Health related quality of life; SF-36, 36-Item Short Form Survey; GDS, Geriatric Depression Scale; MMSE, Mini-Mental State Examination; NW, Nordic Walking; MICT, Moderate-intensity continuous training; HIIT, High-intensity interval training; FSS, Fatigue Severity Scale; MoCA, Montreal Cognitive Assessment; HDS, Hamilton Depression Scale; mMRC, modified medical research council scale; SGRQ, St. George Respiratory Questionnaire; VAS-Visual Analog Scale; FIQ, Fibromyalgia impact questionnaire; MFI, Multidimensional Fatigue Inventory (MFI); BCSB, Brief Cognitive Screening Battery, SVF, Semantic Verbal Fluency; CDT, Clock Drawing Test; FAB, Frontal Assessment Battery; DES-D, Center for Epidemiologic Studies Depression Scale; PROMIS, Patient-reported outcomes measurement information system; SDT, Oral Symbol Digit test from the NIH Toolbox; PCR, Polymerase Chain Reaction; EORTC QLQ-C30, European Organization for Research and Treatment of Cancer Quality of Life; BAI, Beck Anxiety Inventory; POMS-24, Profile of Mood States-24
